# Hounsfield unit change in metastatic abdominal lymph nodes treated with combined hyperthermia and radiotherapy

**DOI:** 10.1371/journal.pone.0318330

**Published:** 2025-03-25

**Authors:** Young Kyu Lee, Kyu Hye Choi, Wonjoong Cheon, Bohyun Kim, In-Ho Kim, Young-nam Kang, HongSeok Jang

**Affiliations:** 1 Department of Radiation Oncology, Seoul St. Mary’s Hospital, College of Medicine, The Catholic University of Korea, Seoul, Republic of Korea; 2 Department of Radiology, Seoul St. Mary’s Hospital, College of Medicine, The Catholic University of Korea, Seoul, Republic of Korea; 3 Division of Medical Oncology, Department of Internal Medicine, Seoul St. Mary’s Hospital, College of Medicine, The Catholic University of Korea, Seoul, Republic of Korea; Affiliated Hospital of Nanjing University of Chinese Medicine: Jiangsu Province Academy of Traditional Chinese Medicine, CHINA

## Abstract

**Objective:**

Hyperthermia has been safely employed in conjunction with low to moderate doses of radiotherapy (RT) to achieve notable tumor responses in cases of previously treated and recurrent malignancies. However, the radiologic monitoring of combined hyperthermia and RT (HTRT) is not extensively documented. This study aimed to assess radiological changes, including alterations in Hounsfield units (HU), in patients undergoing treatment with either HTRT or RT alone for metastatic abdominal lymph nodes.

**Methods:**

CT images were acquired from consecutive 40 patients who received HTRT or RT alone for metastatic abdominal lymph nodes. Target regions were delineated and pre- and post-treatment HU measurements were extracted from these targets. An additional independent t-test was performed to compare the change in mean HU values between the two groups.

**Results:**

The study included 40 patients, 20 patients in the HTRT group and 20 in the RT alone group. In the HTRT group, the average HU after treatment was 58.95 HU, while in the RT-only group, it was 71.42 HU. In the HTRT-treated group, the average HU value of the tumor was lower by 9.05%, with an average of -8.47 HU. (p =  0.011), while in the RT-only treated group, it declined by 0.57% with an average of -0.41 HU.

**Conclusion:**

The HTRT group showed a greater decrease in HU both pre- and post-treatment, indicating a possible indirect marker of tumor necrosis. Sequential trends and survival analyses for comparing the two groups are warranted in subsequent investigations.

## Introduction

Hyperthermia (HT) refers to the intentional administration of elevated temperatures to the human body or specific anatomical regions, to manage various medical conditions. Historical records indicate the utilization of HT since centuries ago. In the 1960s, researchers initiated investigations into the potential use of regulated heat to treat cancer [1]. Dr. Gordon Dewhirst, an American oncologist, conducted pioneering investigations in 1969 concerning the application of HT to potentiate the impact of radiation therapy (RT) on tumor cells [2]. His empirical inquiries established the foundational framework for the integration of HT into oncological therapeutic strategies.

In current medical practice, HT is often used as an adjuvant therapeutic technique in combination with treatments including radiotherapy and chemotherapy [3]. The objective is to augment the efficacy of these interventions by heightening the susceptibility of neoplastic cells to radiation or optimizing pharmaceutical delivery. The combination of HT with RT (HTRT) stands as an established therapeutic approach harnessed in the management of malignancies [4,5]. HT can enhance oxygen levels within the tumor microenvironment. RT manifests superior effectiveness within an oxygen-abundant environment, given that oxygen molecules assume a pivotal role in the generation of reactive oxygen species responsible for inducing DNA damage within malignant cells. Consequently, HT amplifies the potency of RT through the enhancement of tumor oxygenation.

The HTRT is commonly used when cancer has metastasized to the abdominal lymph nodes (LNs) [6,7]. HT augments the susceptibility of neoplastic cells within the LNs to the effects of RT, rendering them more receptive to the injurious impact of ionizing radiation [5]. Abdominal LNs are implicated in a variety of cancer types, including colorectal, ovarian, pancreatic, and gastric cancer [8–11]. The dissemination of cancer cells to the abdominal LNs is identified as regional LN involvement or metastasis. In such instances, the synergistic utilization of HT and RT holds the potential to enhance treatment outcomes.

Necrotic changes in target tissues may occur after HT as it affects the blood flow to the treated area and causes ischemic necrosis [12]. Heat-induced vascular changes reduce blood supply, leading to tissue damage and necrosis. In HT for cancer, inducing local necrosis of tumor tissue is often one of the therapeutic goals. It causes necrotic changes in tumor cells, leading to cell death and tumor destruction. Combining HT with other treatment modalities, such as RT or chemotherapy, further increases the effectiveness of tumor destruction and improves treatment outcomes [5]. Necrosis is the death of cells or tissues and on medical imaging, it is seen as areas of nonviable tissue. Hounsfield Unit (HU) used to observe changes in necrosis are used in computed tomography (CT) scans to measure the radiation density of the tissue and provide useful information about the composition and properties of the imaged structures [13,14]. HU on CT scans allows the detection and evaluation of areas of necrosis and other pathological changes within the body.

In studies evaluating imaging changes, HU values have been used to examine tumor necrosis response to various cancer treatments [15,16]. The study reported by van der Veldt et al. used HU values for the early prediction of clinical outcomes in patients with metastatic renal cell cancer treated with targeted therapies like sunitinib [17]. These criteria (called as Choi criteria) rely on changes in tumor enhancement patterns observed on contrast-enhanced CT scans. They were developed to assess the effectiveness of antiangiogenic therapies, which are known to influence blood flow and vascularity within tumors.

While the clinical benefits of HTRT have been documented, there is limited research examining the radiological changes, particularly HU alterations, in metastatic lymph nodes treated with this combined approach. Understanding these imaging biomarkers could provide valuable insights into treatment response assessment and potentially guide therapeutic decision-making. In this study, necrotic changes in abdominal LNs after HTRT were evaluated by CT before and after treatment and compared with changes with conventional RT alone.

## Materials and Methods

CT scans were acquired prior to and subsequent to treatment in a cohort of consecutive 40 patients undergoing either Combined HTRT or RT alone for metastatic abdominal LNs from 01/01/2019 to 31/03/2022 at Seoul St. Mary’s Hospital. The decision between HTRT and RT alone was made based on several factors including: previous radiation history to the treatment area, patient’s performance status, and feasibility of delivering conventional radiation doses. HTRT was primarily selected for cases where conventional full-dose RT was challenging due to prior radiation exposure or compromised performance status.

The REMISSION 1°C device (AdipoLABs, Seoul, Republic of Korea), which was employed for hyperthermia, has a medical high-frequency thermogenic instrument designed to augment cancer therapy through the generation of potent deep-seated heat, operating at a high frequency of 0.46MHz to elevate internal body temperatures. Target delineation was generated with MIM 7.1.9 workstation (MIM Software Inc., USA), and subsequent HU measurements were derived from the designated target regions.

Using 64-detector row CT scanners (Somatom Sensation 64; Siemens Healthineers or Discovery CT750 HD; GE Healthcare) or a 128-channel CT scanner (Somatom Definition AS + ; Siemens Healthineers), contrast-enhanced CT images were acquired. The CT protocols were as follows: 5-mm section thickness, 100-200 mAs with automated tube current modulation, 100-120 kVp.

For every individual in the cohort, the average HU value within the tumor region was computed from both the pre- and post-treatment CT scans. The average HU values were determined independently for each group. The null hypothesis (H0) is that no disparity exists in average HU values between the HTRT and RT-alone groups. Subsequently, a 2-sample Student’s t-test was employed to assess whether there was a statistically significant difference in average HU values between the two groups.

The correlation between HU changes and various clinical variables including treatment modality (HTRT vs. RT alone), radiation dose, patient age, sex, and primary tumor site was further evaluated. These variables were selected based on their potential influence on treatment response and tissue density changes. We investigated the correlation between variables and HU values using a linear regression model To determine whether the HU change between the two groups was statistically significant, we examined the p-value associated with the coefficient. A p-value lower than a specific significance threshold of 0.05 implied that a discrepancy in HU values between the two groups. The study was approved by Seoul St. Mary’s Hospital institutional review board (No: KC23RISI0567). Informed consent was waived because of the retrospective nature of the study and the analysis used anonymous clinical data. Data were accessed for research purposes in 27/07/2024.

## Results

The characteristics of the 20 patients who underwent combined HTRT and the 20 patients who underwent RT alone are summarized in Table 1. No statistically significant differences in the clinical characteristics were observed between the two groups. However, the effective biological dose of the therapeutic radiation was notably lower in the HTRT group. The HTRT combination was applied in situations where complete irradiation with radiation therapy is challenging, such as areas previously treated to radiation or in patients with compromised performance status. The objective of this study was to evaluate the imaging effects caused by combined relatively lower dose radiation and hyperthermia therapy compared to the conventional dose of RT alone. Notably, no statistical differences were noted in the average HU value prior to treatment or the volume of the treated region.

**Table 1:** Patient characteristics.

Characteristics		HTRT group N (%) or median (range)	RT alone group N (%) or median (range)	p-value
Sex	Male	16 (80)	15 (75)	0.705
	Female	4 (20)	5 (25)	
Age (years)		64 (26–87)	59 (38–79)	0.141
Primary site	Upper GI	3 (15)	2 (10)	0.244
	Lower GI	4 (20)	8 (40)	
	Hepatobiliary	11 (55)	5 (25)	
	Genitourinary	2 (10)	4 (20)	
	Unknown origin	0	1 (5)	
RT fractional dose (cGy)	180	18 (90)	12 (60)	0.102
	200	1 (5)	5 (25)	
	250	1 (5)	2 (10)	
	300	0	1 (5)	
RT total dose (cGy)		5310 (2520–5600)	5580 (3000–5940)	0.085
BED10 (cGy)		6311 (2974–6720)	6584 (3900–7009)	0.046
HT number (fractions)		9 (4–20)	NA	NA
Pretreatment target volume (cm^3^)		38.6 (2.1–111.6)	35.1 (1.4–213.8)	0.094
Pretreatment average HU (HU)		73.8 (41.1–117.7)	63.7 (40.9–118.6)	0.537

HTRT, combined hyperthermia-radiotherapy; RT, radiation therapy; GI, gastrointestinal; BED10, biologically effective dose when α/β ratio is 10; HT, hyperthermic therapy; HU, Hounsfield units

In this study, average HU values and changes in tumor volume were calculated for 20 patients treated with HTRT and 20 patients treated with RT alone. The median value of the average HU of each patient after treatment in the HTRT group was 58.95 HU (range: 15.03 – 136.57 HU) and the median value of the average HU after treatment in the RT-only group was 71.42 HU (range: 37.53 – 144.41 HU). The average change in HU value of the tumor was reduced by 9.05% (range: –80.30% ± 29.94%) with a median value of –8.47 HU in the HTRT-treated group, whereas it decreased by 0.57% (range: –23.14% ± 71.03%) with a median value of –0.41 HU in the RT-only treated group. (p =  0.011).

**Fig 1:**
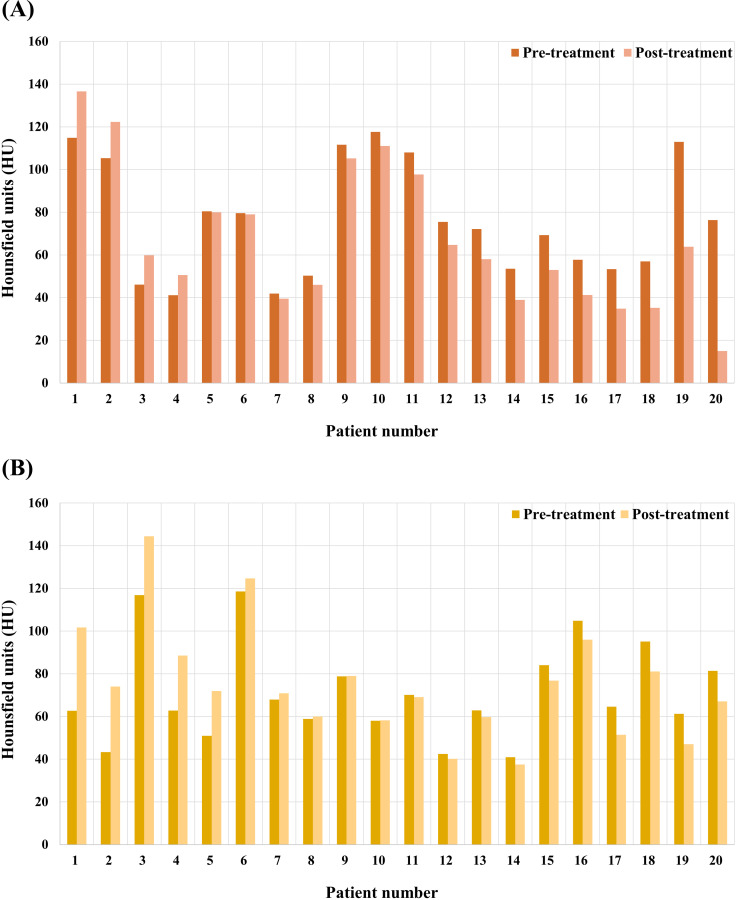
Average Hounsfield units of pre and post-treatment in target lesion for patients treated with combined hyperthermia-radiotherapy (A) and radiotherapy alone (B) group.

**Fig 2:**
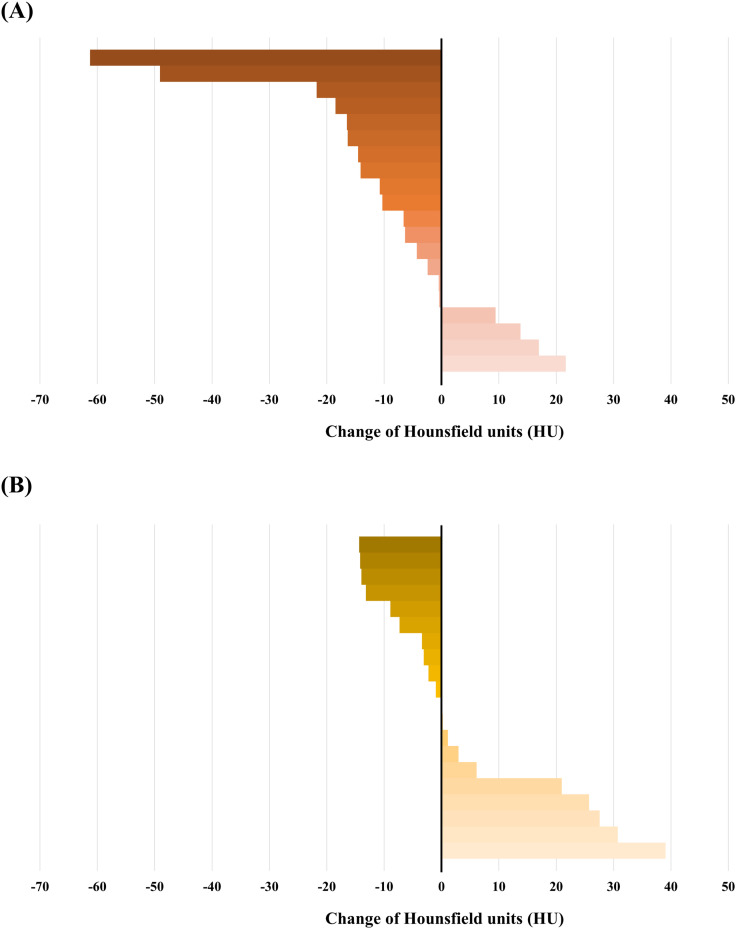
Change of average Hounsfield units in target lesion for patients treated with combined hyperthermia-radiotherapy (A) and radiotherapy alone **(B)**.

**Fig 3:**
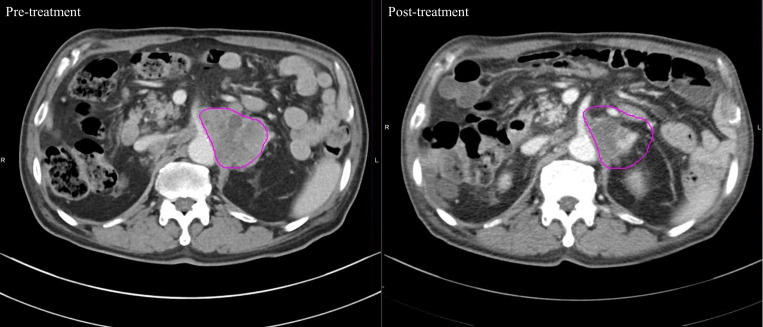
Clinical features with good radiological response in the combined hyperthermia-radiotherapy group.

Fig 1 shows a graphical representation of the HU values before and after treatment for each of the 40 patients. Changes observed before and after each treatment are depicted in Fig 2. The number of patients who showed reduced HU values in the HTRT group (16 patients, 80%) was greater than that in the RT alone group (10 patients, 50%). Moreover, the degree of reduction was significantly greater in the HTRT group. Fig 3 depicts clinical features of good radiologic response in the combined hyperthermia-radiotherapy group.

Using a linear regression model, we investigated the relationship between treatment modalities and changes in HU values between with and without combined hyperthermic therapy, Changes in HU values showed statistically significant differences (p =  0.023). On the other hand, the alteration in HU value did not result in a statistically different radiation dose (p =  0.237). The detailed values of linear regression between HU changes and treatment modality and demographic characteristics are summarized in Table 2.

**Table 2:** Linear regression analysis.

Variable	Regression coefficient	Standard deviation	p-value
Sex	–3.079	10.679	0.775
Age	0.016	0.344	0.963
Primary site	–2.153	4.622	0.644
RT total dose	–0.007	0.006	0.237
BED10	–0.005	0.005	0.327
HT	19.811	8.330	0.023

RT, radiation therapy; BED10, biologically effective dose when α/β ratio is 10; HT, hyperthermic therapy

## Discussion

This study aimed to evaluate radiological changes, specifically HU alterations, in metastatic abdominal lymph nodes treated with HTRT compared to RT alone. Our findings demonstrated that the HTRT group showed a significantly greater decrease in HU values (9.05% reduction) compared to the RT alone group (0.57% reduction), suggesting more pronounced necrotic changes in the combined treatment group. When high-frequency current is energized in the human body, heat is generated in tissues. This is called ‘deep-seated heat’ [18]. This is because when high-frequency electrical energy is applied, whenever the direction of the current changes, the molecules constituting the tissue vibrate and rub against each other, generating bioheat through rotational motion, twisting motion, and collision motion. Unlike other types of current, high-frequency current, which does not stimulate sensory and motor nerves, can heat specific areas in body tissues without causing muscle contraction [19]. The high-frequency energy converted into biothermal energy raises the temperature of tissues to improve cell function and increase blood flow. The generally known function recovery temperature of the tissue is 42°C [20,21].

The European Society of Hyperthermic Oncology (ESHO) has established quality standards for hyperthermia interventions. A pivotal criterion entails direct temperature measurement within the tumor, ensuring validation of targeted volume heating to the specified range of 40-43°C [22,23]. When the local temperature of the tissue rises above 40°C, arterial and capillary dilation occurs by direct effect, blood flow is increased, the body’s defense mechanism, blood circulation is promoted, and metabolism is enhanced. The increase in capillary blood flow due to deep heat generation is 4-5 times higher than at rest [24]. In addition, the supply of oxygen, nutrients, antibodies, and leukocytes increases, and the hydrostatic pressure of capillaries increases due to vasodilation, so lymphatic circulation is promoted.

The results of this study showed that, during combined HTRT, the progression of necrosis was more pronounced, showing a greater reduction in HU. The combination of HTRT can have a synergistic effect on tumor tissue, potentially resulting in necrotic changes. HT can sensitize tumor cells to the effects of RT [25]. When cells are exposed to both high heat and radiation, the combined stresses destroy cell structure and function more effectively than either treatment alone. This can lead to more extensive cellular damage and in some cases necrosis. HT increases blood flow to the tumor site and potentially improves oxygen delivery. Improved oxygenation with RT intensifies cell damage by enhancing the formation of free radicals (reactive oxygen species) produced by radiation. When oxygen demand exceeds supply, ischemia occurs, contributing to necrotic changes. The combination of HTRT stimulates the immune system’s response to tumor antigens released as a result of cell damage [26,27]. This immune response targets and eliminates damaged cells, causing necrotic changes at the treatment site.

Several studies have shown that necrotic tumors, as indicated by low HU values on pretreatment CT, have a poor prognosis [28,29]. However, there are few studies analyzing the changes in HU among different treatment methods. Our study showed that combining hyperthermia with a relatively low radiation dose resulted in a dramatic reduction in HU, which is likely to induce more necrosis. A response is often characterized by at least a 10 HU decrease in tumor attenuation, and Choi criteria had a significantly better predictive value for progression-free survival and overall survival in partial response patients [17].

The combination of HTRT can increase cellular stress, leading to cell death (programmed cell death). In case of severe stress or inadequate repair mechanisms, cells die. HT and RT can affect the tumor microenvironment, by promoting inflammation and disrupting the balance of signaling molecules. These changes contribute to necrotic processes within the tumor. This is a mechanism that is expected to eventually result in good tumor control [30,31].

Furthermore, during clinical follow-up, patients in the HTRT group showed notable improvements in quality of life, particularly regarding abdominal pain reduction and improved mechanical obstruction. Although these clinical benefits were not systematically evaluated in this study, these observations suggest potential symptomatic advantages of HTRT that warrant further investigation using standardized assessment tools. Pain and symptom control is also an important clinical goal in metastatic cancer patients who require palliative treatment.

These are the major limitations of our study. First, the retrospective nature of the study may introduce inherent selection bias. However, we attempted to minimize this through consecutive patient sampling and matching key clinical characteristics between groups. Second, While our sample size was relatively small (n =  40), our findings showed statistically significant differences between groups, and power calculations suggest this was adequate for our primary endpoint. However, a larger prospective study would help validate these results.

This study is meaningful in that it shows the actual clinical results of combined HTRT. Notably, future studies on the long-term control of local recurrence and survival analysis of combined HTRT have been planned. In conclusion, this study demonstrated that HTRT induces greater reductions in HU values compared to RT alone in metastatic abdominal lymph nodes, suggesting enhanced necrotic changes. These findings provide radiological evidence for the potential benefits of adding hyperthermia to radiotherapy in selected cases. Future prospective studies with larger cohorts and longer follow-up are warranted to validate these findings and correlate them with clinical outcomes.
